# Influence of COVID-19 pandemic on the distribution and drug resistance of pathogens in patients with bloodstream infection

**DOI:** 10.3389/fpubh.2025.1607801

**Published:** 2025-07-28

**Authors:** Yuanyuan Liu, Hui Song, Yanli Wu, Lihua Liu, Ning Li, Min Zhang, Yusen Li, Xiujuan Meng

**Affiliations:** ^1^Clinical Laboratory, Affiliated Hospital of Jining Medical University, Jining, China; ^2^Healthcare-Associated Infection Control Department, Affiliated Hospital of Jining Medical University, Jining, China

**Keywords:** coronavirus disease 2019, COVID-19, pandemic, bloodstream infection, pathogen distribution, drug resistance

## Abstract

**Background:**

The pandemic of Coronavirus Disease 2019 (COVID-19) significantly impacted healthcare systems worldwide, especially improving awareness of infection prevention and control in medical institutions. However, it remains unclear to what extent COVID-19 influenced the occurrence of bloodstream infection (BSI). This study aimed to analyze the distribution and antibiotic resistance patterns of pathogens responsible for BSI before and after the COVID-19 pandemic in a tertiary hospital.

**Methods:**

Pathogens from patients with BSI were collected from January 2018 to December 2022. Pathogen identification was performed using matrix-assisted laser desorption/ionization time-of-flight mass spectrometry (MALDI-TOF MS). Antimicrobial susceptibility testing was conducted using broth microdilution, the Kirby-Bauer (K-B) disk diffusion method, and Etest. Data were analyzed using WHONET and SPSS software. This study was approved by the Medical Ethics Research Committee of the hospital (2023-11-C026).

**Results:**

Following the COVID-19 pandemic, the blood culture submission rate decreased from 12.82 to 11.07%, while the standardized blood culture positivity rate increased from 0.53 to 0.62%. Among the identified pathogens, Gram-negative bacteria accounted for 67.90%, Gram-positive bacteria for 28.82%, and fungi for 3.28%. The most frequently isolated pathogens were *Escherichia coli*, *Klebsiella pneumoniae*, and *Staphylococcus aureus*. The resistance rate of *E.coli* to ciprofloxacin increased from 60.10 to 66.84% post-pandemic, whereas *K. pneumoniae* showed a reduction in cefepime resistance, decreasing from 25.42 to 15.54%. Additionally, the proportion of extended-spectrum beta-lactamase (ESBL)-producing *E. coli* increased from 35.93 to 50.63%. In contrast, *S. aureus* exhibited no significant changes in resistance to commonly used antibiotics post-pandemic.

**Conclusion:**

The COVID-19 pandemic impacted the distribution and antibiotic resistance of pathogens in patients with BSI. Notably, the prevalence of ESBL-producing *E. coli* were increased, while the isolation rates of other multidrug-resistant organisms remained relatively stable.

## 1 Introduction

Bloodstream infection (BSI) is a prevalent cause of both community-acquired and healthcare-associated infections. Each year, BSI accounts for an estimated 1.2 million cases and 157,000 deaths in Europe, and between 575,000 and 677,000 cases and 79,000 to 94,000 deaths in North America, representing a growing public health concern globally (1). BSI not only leads to substantial short-term morbidity, occurring at rates from 1.3 to 31.2 episodes per 1,000 hospital admissions, with mortality rates ranging from 10.6 to 22.7% (2), but is also linked to poor long-term outcomes extending 1 year or more post-infection (3, 4). Rapid identification of the causative pathogen and timely initiation of appropriate antimicrobial therapy are critical for improving patient outcomes and reducing the healthcare burden associated with BSI.

Antimicrobial resistance has emerged as one of the most serious threats to global health in recent years, complicating both prevention and treatment of patients with BSI. The incidence of BSI caused by antibiotic-resistant organisms, particularly multidrug-resistant Gram-negative bacteria, is on the rise (5, 6). The global pandemic of COVID-19 in late 2019 placed unprecedented pressure on healthcare systems. SARS-CoV-2 infections increased patients’ susceptibility to bacterial co-infections, significantly elevating the risk of BSI and sepsis (7). The pandemic also had complex implications for antibiotic resistance, with some studies reporting a rise in multidrug-resistant bacterial infections, particularly in intensive care units (ICUs) (8–12), while others did not observe an increase in multidrug-resistant infections (13, 14). Multiple factors contributed to the varying trends in antibiotic resistance during the COVID-19 pandemic (8, 15), and experts suggested that the pandemic’s impact on resistance patterns had only become apparent over time as more data were gathered (16). Notably, changes in antibiotic resistance largely depended on the setting, with considerable variability across departments, hospitals, and countries, underscoring the importance of analyzing existing surveillance data (15).

This study evaluates the impact of the COVID-19 pandemic on pathogen distribution and antimicrobial resistance patterns in BSI in a tertiary hospital, comparing pre-pandemic (2018–2019) and pandemic (2020–2022) periods. These findings will provide a foundation for future diagnosis, treatment, and prevention of BSI.

## 2 Methods

### 2.1 Study design and population

This study was a five-year retrospective analysis of clinical and laboratory data from patients with BSI in a tertiary teaching hospital, covering the period from January 2018 to December 2022. This hospital has 4,100 beds and 5,254 healthcare workers, with the updating and development of disciplines, the hospital currently has 82 clinical departments and 15 intensive care units, admitted an average of 197,076 patients annually and served about 20 million residents in Southwest Shandong Province. Contaminating pathogens and duplicate strains detected in the same patient during a single hospitalization were excluded. All patient identifiers, including names, ID numbers, and identity card details, were anonymized to protect patient privacy. Data were categorized into two groups: the pre-pandemic cohort (January 2018–December 2019) and the pandemic cohort (January 2020–December 2022). This study was approved by the Medical Ethics Research Committee of the Affiliated Hospital of Jining Medical University (2023-11-C026).

### 2.2 Bacterial culture and identification

The collection, transport, and culture of blood specimens followed the guidelines outlined in the *National Clinical Inspection Operating Procedures* (17). Bacterial identification was performed using matrix-assisted laser desorption/ionization time-of-flight mass spectrometry. Quality control was ensured using standard strains, including *Staphylococcus aureus* (*S. aureus*) ATCC29213, *Streptococcus pneumoniae* ATCC49619, *Escherichia coli* (*E. coli*) ATCC25922, and *Pseudomonas aeruginosa* ATCC27853, which were obtained from the Clinical Laboratory Center of the National Health and Family Planning Commission. All clinical specimens were subjected to initial phenotypic identification upon arrival. Representative bacterial isolates were cryopreserved at −80°C in 15% glycerol-supplemented medium and maintained in the institutional strain collection.

### 2.3 Antimicrobial susceptibility test

Antimicrobial susceptibility testing was conducted using the VITEK2 Compact automated analyzer and the Kirby-Bauer disk diffusion method. Results that appeared ambiguous were confirmed using Etest strips. Antimicrobial susceptibility interpretations followed the guidelines of the Clinical Laboratory Standards Institute (18).

### 2.4 Definitions

Two blood culture bottles collected from the same limb site or puncture location were considered a single set, with one positive bottle in the set deemed sufficient for a positive blood culture result. The blood culture submission rate was defined as the number of blood culture sets submitted divided by the total number of all culture samples submitted. The standardized blood culture positivity rate was calculated using the direct standardization method, with the pooled population of hospitalized patients across all study years (2018–2022) serving as the standard population. The antimicrobial resistance rate was calculated as the number of patients with resistant pathogens in blood cultures divided by the total number of patients with specified pathogens in blood cultures. The antimicrobial use density (AUD) is expressed as the defined daily doses (DDDs) of antimicrobial agents consumed per 100 inpatient-days.

### 2.5 Statistical analysis

WHONET software (version 5.6) was used to analyze pathogen antimicrobial resistance patterns. Statistical analyses were conducted using SPSS software (version 26.0). Chi-square test, Fisher’s exact test or Mann–Whitney U test were used appropriately to compare the differences between the pre-pandemic and pandemic periods. Spearman correlation analysis was performed to assess the relationship between antimicrobial use density and bacterial resistance rates. The *p*-value of less than 0.05 was considered statistically significant.

## 3 Results

### 3.1 Blood culture submission and positivity rate

The blood culture submission rates during 2018–2022 were as follows: 14.07% (25,463/181,031), 11.72% (23,815/203,278), 11.06% (20,316/183,694), 11.97% (25,932/216,546), and 10.09% (20,269/200,832). During the pre-pandemic period (2018–2019), the rate was 12.82% (49,278/384,309), declining significantly to 11.07% (66,517/601,072) during the pandemic years (2020–2022) (*p* < 0.05). Statistical analysis revealed a significant downward trend in the proportion of blood cultures submitted during the pandemic (*p* < 0.05).

**Figure 1:**
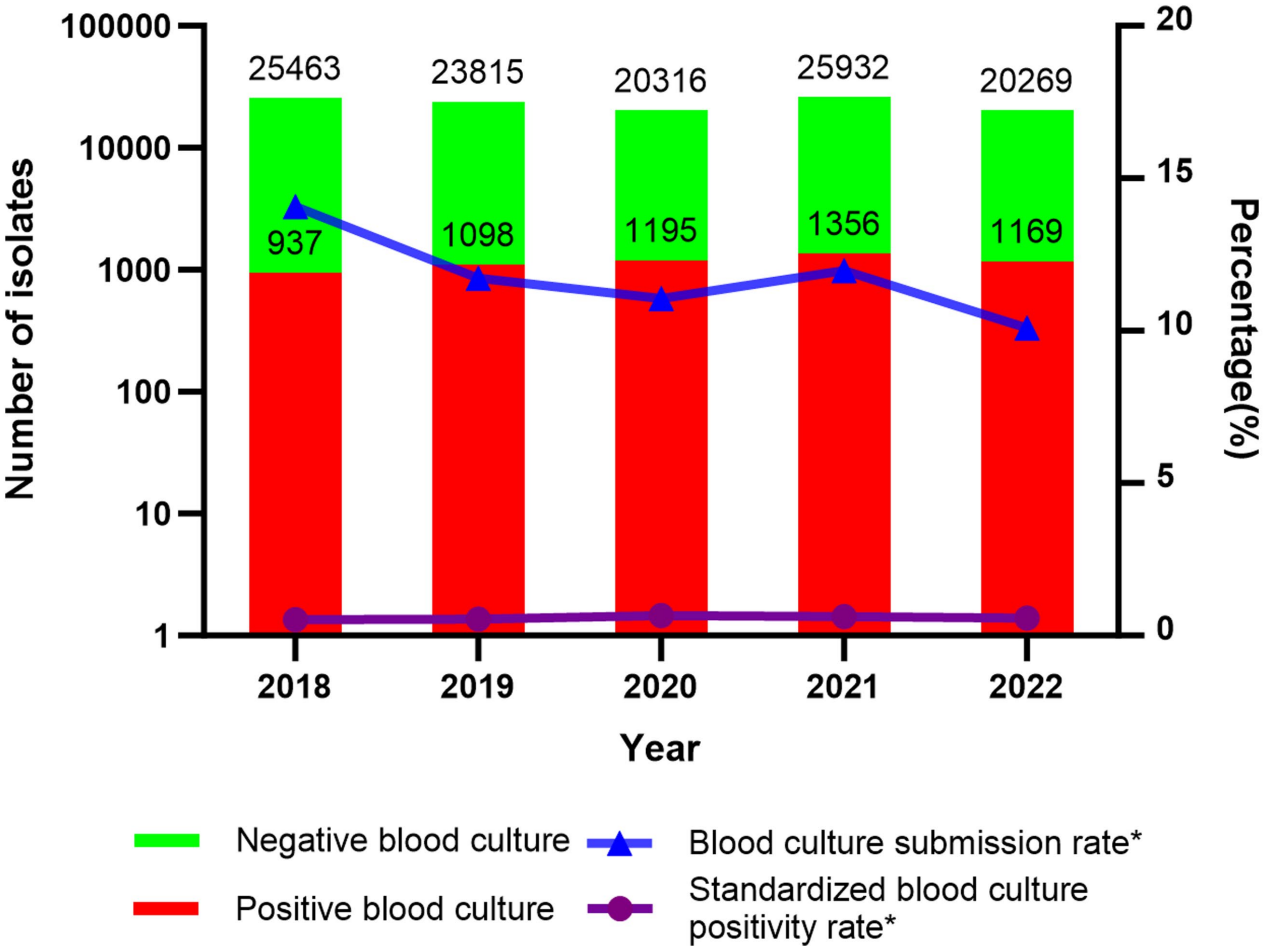
Number of negative/positive blood cultures, submission rate, and standardized positivity rate; *p < 0.05.

The standardized blood culture positivity rates over 5 years were as follows: 0.52% (937/181,031), 0.54% (1,098/203,278), 0.65% (1,195/183,694), 0.63% (1,356/216,546), and 0.58% (1,169/200,832). The standardized blood culture positivity rate before the pandemic was 0.53% (2,035/384,309), while during the pandemic, it increased to 0.62% (3,720/601,072). Statistical analysis showed a significant increase in the standardized blood culture positivity rate during the pandemic compared to the pre-pandemic period (*p* < 0.05) (Figure 1).

### 3.2 Clinical characteristics of patients with positive blood culture

**Figure 2:**
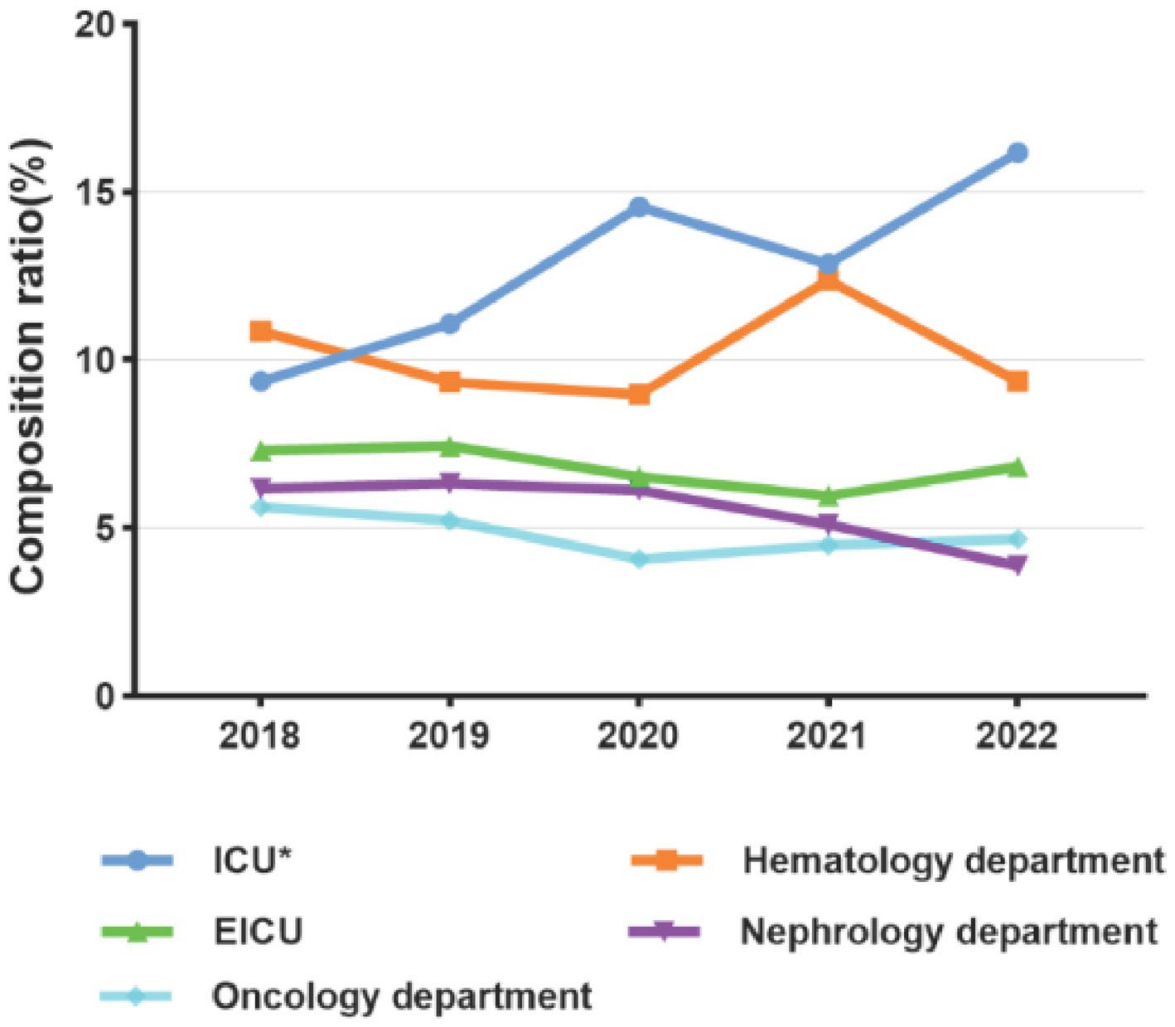
Composition of main departments with BSI patients before (2018–2019) and during the COVID-19 pandemic (2020–2022); *p < 0.05.

The number of patients with positive blood cultures from 2018 to 2022 were as follows: 534, 632, 735, 824, and 758. The male-to-female ratio was 1.26: 1. Using 14 years as a cut-off age, patients were categorized into an adult group and a pediatric group. The median age of patients in the adult group was 62 years (range: 50–72), while the median age in the pediatric group was 0.92 years (range: 0.07–3). The top five departments with the highest number of BSI patients were ICUs, hematology department, emergency ICU (EICU), nephrology department, and oncology department. The proportion of ICU patients among all blood culture-positive patients significantly increased from 10.29% (120/1,166) before the COVID-19 pandemic to 14.42% (334/2,317) during the pandemic (*p* < 0.05). There were no significant differences in the composition ratios of other departments before and during the pandemic (Figure 2).

### 3.3 Strain composition

The pathogens identified in blood cultures from 2018 to 2022 included Gram-negative bacteria, Gram-positive bacteria, and fungi, with Gram-negative bacteria accounting for 67.90% (1,988/2,928), Gram-positive bacteria for 28.82% (844/2,928), and fungi for 3.28% (96/2,928). Among the Gram-negative bacteria, the highest isolation rate was *E. coli* [36.65% (1,073/2,928)], followed by *K. pneumoniae* [11.20% (328/2,928)] and *Pseudomonas aeruginosa* [4.68% (137/2,928)]. Among the Gram-positive bacteria, *S. aureus* had an isolation rate of 5.33% (156/2,928), followed by coagulase-negative staphylococci. Among the fungi, *Candida tropicalis* was the predominant species. When comparing the isolation rates of major pathogens in BSI, no significant differences were observed in most pathogens (all *p* > 0.05). However, the isolation rate of *Brucella melitensis* (*B. melitensis*) was significantly lower during the pandemic compared to pre-pandemic levels (2.93% vs. 4.47%, *p* = 0.027), as was the isolation rate of *Acinetobacter baumannii* (*A. baumannii*) (0.92% vs. 2.02%, *p* = 0.011). Pathogens isolated from patients with BSI before and during the COVID-19 pandemic were provided in Table 1.

**Table 1:** Pathogens isolated from patients with BSI before and during the COVID-19 pandemic [*n* (%)].

Pathogens	Pre-pandemic (2018–2019)		Pandemic (2020–2022)			*p-*value
	2018	2019	2020	2021	2022	
Gram-negative bacteria	393 (71.59)	423 (66.41)	351 (65.49)	420 (66.46)	401 (69.86)	0.386
*Escherichia coli*	217 (39.53)	220 (34.54)	191 (35.63)	232 (36.71)	213 (37.11)	0.853
*Klebsiella pneumoniae*	55 (10.02)	63 (9.89)	70 (13.06)	68 (10.76)	72 (12.54)	0.0076
*Pseudomonas aeruginosa*	32 (5.83)	33 (5.18)	20 (3.73)	22 (3.48)	30 (5.23)	0.090
*Brucella melitensis*	26 (4.74)	27 (4.24)	11 (2.05)	17 (2.69)	23 (4.01)	0.027*
*Acinetobacter baumannii*	10 (1.82)	14 (2.20)	5 (0.93)	6 (0.95)	5 (0.87)	0.011*
*Salmonella*	13 (2.37)	7 (1.10)	12 (2.24)	3 (0.47)	7 (1.22)	0.344
*Enterobacter cloacae*	9 (1.64)	5 (0.78)	9 (1.68)	9 (1.42)	3 (0.52)	0.951
Other negative bacteria	31 (5.65)	54 (8.48)	33 (6.16)	63 (9.97)	48 (8.36)	-
Gram-positive bacteria	140 (25.50)	195 (30.61)	166 (30.97)	191 (30.22)	152 (26.48)	0.568
*Staphylococcus aureus*	29 (5.28)	26 (4.08)	38 (7.09)	42 (6.65)	21 (3.66)	0.170
*Staphylococcus hominis*	15 (2.73)	37 (5.81)	16 (2.99)	24 (3.80)	15 (2.61)	0.082
*Staphylococcus epidermidis*	12 (2.19)	28 (4.40)	14 (2.61)	25 (3.96)	25 (4.36)	0.665
*Enterococcus faecium*	11 (2.00)	18 (2.83)	22 (4.10)	18 (2.85)	16 (2.79)	0.223
*Streptococcus pneumoniae*	21 (3.83)	13 (2.04)	8 (1.49)	15 (2.37)	8 (1.39)	0.050
*Staphylococcus haemolyticus*	5 (0.91)	15 (2.35)	7 (1.31)	13 (2.06)	7 (1.2)	0.773
*Enterococcus faecalis*	13 (2.37)	7 (1.10)	8 (1.49)	10 (1.58)	8 (1.39)	0.679
*Streptococcus mitis*	9 (1.64)	7 (1.10)	11 (2.05)	7 (1.11)	10 (1.74)	0.573
Other positive bacteria	25 (4.55)	44 (6.91)	42 (7.84)	37 (5.85)	42 (7.32)	-
Fungi	16 (2.91)	19 (2.98)	19 (3.54)	21 (3.32)	21 (3.66)	0.411
*Candida tropicalis*	8 (1.46)	5 (0.78)	5 (0.93)	11 (1.74)	8 (1.39)	0.503
Other fungi	8 (1.46)	12 (1.88)	12 (2.24)	10 (1.58)	13 (2.26)	-
Total	549 (100.00)	637 (100.00)	536 (100.00)	632 (100.0)	574 (100.00)	-

*p < 0.05.

### 3.4 Detection of multidrug-resistant bacteria

**Figure 3:**
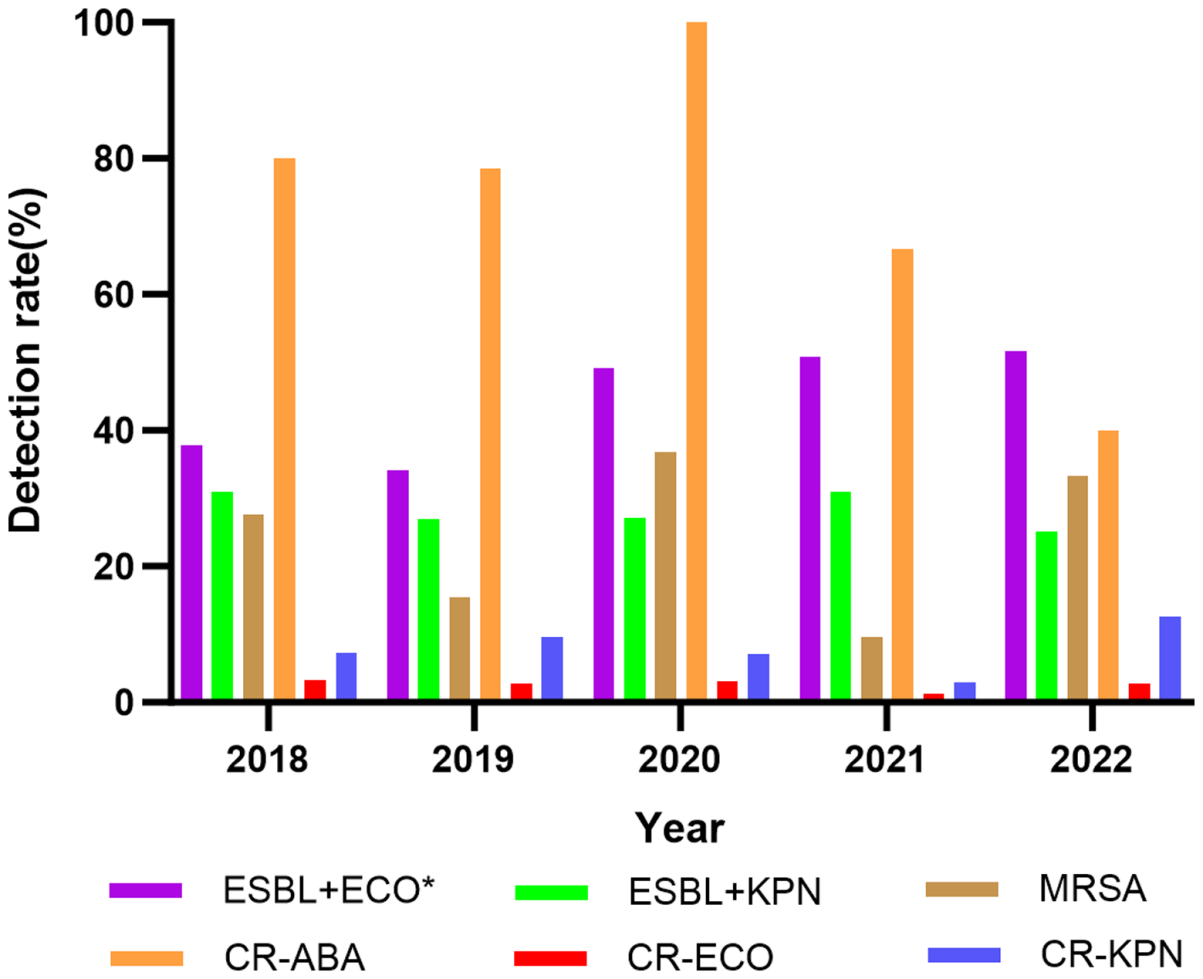
Changes of the detection rate of multidrug-resistant bacteria in BSI, 2018–2022. ESBL+ECO, ESBL+*E. coli*; ESBL+KPN, ESBL+*K. pneumoniae*; MRSA, methicillin-resistant *Staphylococcus aureus*; CR-ABA, carbapenem-resistant *A. baumannii*; CR-ECO, carbapenem-resistant *E. coli*; CR-KPN, carbapenem-resistant *K. pneumoniae*; *p < 0.05.

From 2018 to 2022, the number of multidrug-resistant bacteria identified in BSI were as follows: 129, 121, 143, 153, and 155. These pathogens primarily included *ESBL*-producing *E. coli* (ESBL+*E. coli*), *ESBL*-producing *K. pneumoniae* (ESBL+*K. pneumoniae*), methicillin-resistant *Staphylococcus aureus* (MRSA), carbapenem-resistant *A. baumannii*, carbapenem-resistant *K. pneumoniae*, and carbapenem-resistant *E. coli*. The detection rates of multidrug-resistant bacteria showed only minor variation before and during the COVID-19 pandemic. For instance, the detection rate of *ESBL+ E. coli* increased significantly from 35.93% (157/437) to 50.63% (322/636) (*p* < 0.05). However, there were no significant differences in the detection rates of other multidrug-resistant bacteria (*p* > 0.05) (Figure 3).

### 3.5 Resistance of major pathogens in BSI to common antibiotics

The resistance rates of *E. coli* isolated from BSI to amikacin, carbapenems (ertapenem, meropenem, and imipenem), piperacillin/tazobactam, and cefotetan were all less than 7%. The resistance rates to tobramycin and third- and fourth-generation cephalosporins (cefepime and ceftazidime) ranged from 10 to 30%. Resistance rates to penicillins (ampicillin), first-, second-, and third-generation cephalosporins (cefazolin, cefuroxime, and ceftriaxone), sulfonamides (trimethoprim-sulfamethoxazole/cotrimoxazole), and quinolones (ciprofloxacin and levofloxacin) generally exceeded 50%. When comparing the resistance rates to common antibiotics, we found that the resistance rate to ciprofloxacin was significantly higher during the COVID-19 pandemic (66.84% vs. 60.10%, *p* < 0.05). No significant differences were observed in the resistance rates to other antibiotics.

The resistance rates of *K. pneumoniae* to amikacin, carbapenems (ertapenem, meropenem, and imipenem), and cefotetan were all lower than 15%. The resistance rates to piperacillin/tazobactam and tobramycin were below 20%, while the resistance rates to third- and fourth-generation cephalosporins (ceftazidime and cefepime), levofloxacin, and gentamicin were less than 35%. Resistance rates to first-, second-, and third-generation cephalosporins (cefazolin, cefuroxime, and ceftriaxone) and ampicillin/sulbactam were relatively higher, ranging from 35 to 51%. Among the common antibiotics, we found that the resistance rate to cefepime was significantly lower during the pandemic (15.54%) compared to before the pandemic (25.42%), with this difference being statistically significant (*p* < 0.05). No significant differences were observed in the resistance rates to other antibiotics.

**Figure 4:**
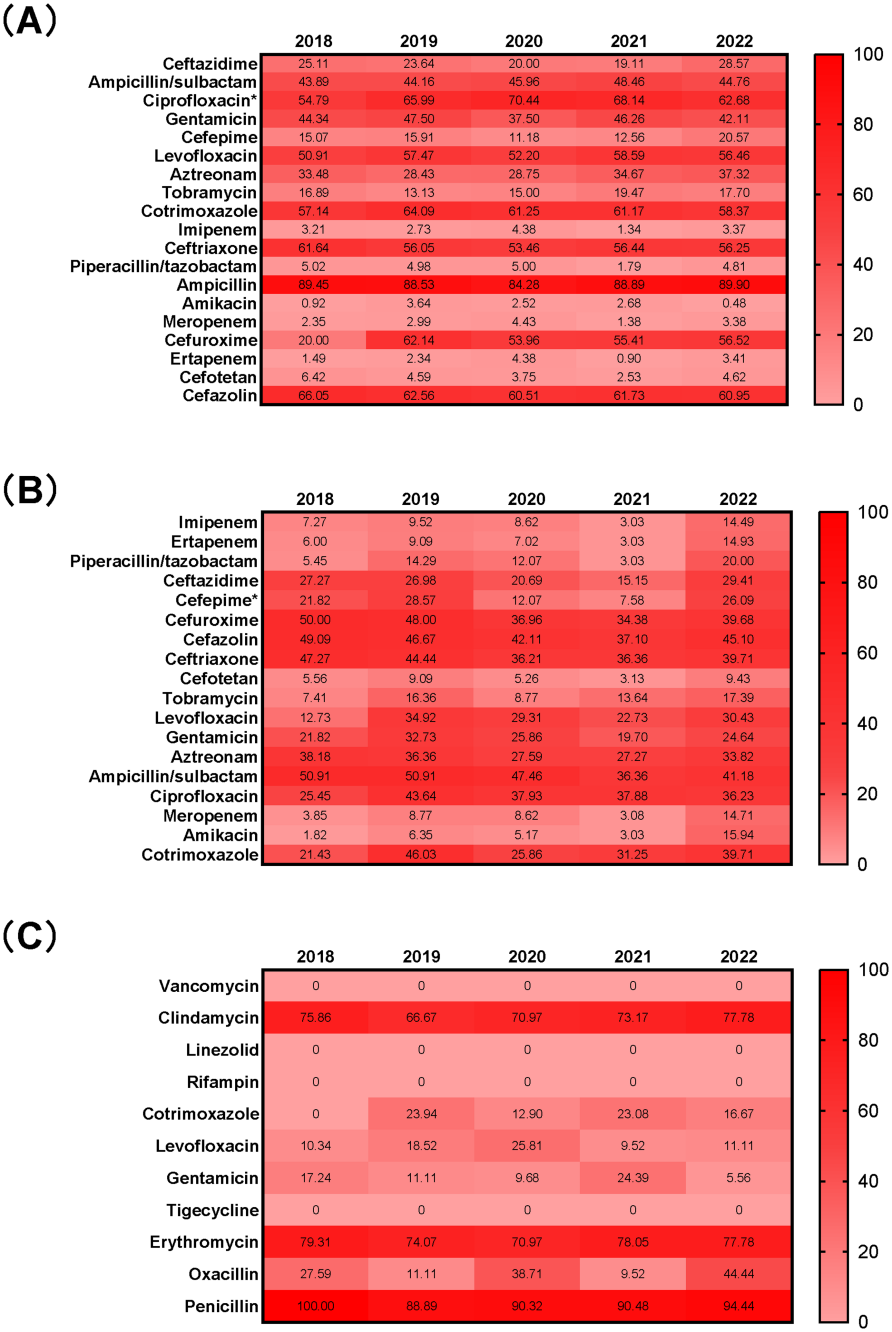
Antimicrobial resistance profiles of *E. coli* **(A)**, *K. pneumoniae* **(B)**, and *S. aureus* **(C)** isolated from blood from 2018 to 2022 Numbers in the heat maps represented the percentage of antimicrobial resistance. *p < 0.05 when compared to data before and during the COVID-19 pandemic.

*S. aureus* showed no resistance to linezolid, rifampicin, tigecycline, or vancomycin over the five-year period. The resistance rates to levofloxacin, gentamicin, and cotrimoxazole were all below 26%, while the resistance rates to clindamycin and erythromycin were higher than 67%. The resistance rate to penicillin exceeded 88%. No significant differences were found in the resistance rates of *S. aureus* to any antibiotics before and during the pandemic (Figure 4).

### 3.6 Correlation between AUD and resistance rate

While antimicrobial use density in inpatients showed a consistent decline from 2018 to 2022 (43.57 to 36.81 DDDs/100 patient-days), no statistically significant difference was observed between pre-pandemic and pandemic periods (*p* > 0.05). The carbapenems, imipenem and meropenem AUD showed no significant differences (*p* > 0.05), with the former two demonstrating a declining trend and the latter an upward trend (Table 2). No correlation was found between imipenem/meropenem/ carbapenems use density and resistance rates of *E. coli*/*K. pneumoniae* to these agents in BSI (*p* > 0.05) (Table 3).

**Table 2:** Comparison of carbapenem use density before and during the COVID-19 pandemic (DDDs/100 patient-days).

Antibiotic agents	Pre-pandemic (2018–2019)		Pandemic (2020–2022)			*p-*value
	2018	2019	2020	2021	2022	
Imipenem	0.62	0.69	0.65	0.74	0.79	0.248
Meropenem	1.37	1.14	0.81	0.93	0.98	0.083
Carbapenems	2.12	1.58	1.46	1.66	1.78	0.564

Carbapenems included imipenem and meropenem.

**Table 3:** Correlation between imipenem/meropenem/carbapenems use density and resistance rates to carbapenems in *E. coli, K. pneumoniae.*

Antimicrobial resistance rate	Imipenem		Meropenem		Carbapenems	
	*r*	*p-*value	*r*	*p-*value	*r*	*p-*value
*E.coli—*imipenem	−0.200	0.747			−0.200	0.747
*E.coli*— meropenem			−0.400	0.505	−0.500	0.391
*K. pneumoniae—*imipenem	0.400	0.505			−0.100	0.873
*K. pneumoniae*—meropenem			0.100	0.873	−0.100	0.873

Carbapenems included imipenem and meropenem.

## 4 Discussion

In our study, the blood culture submission rate significantly declined from 12.82% in the pre-pandemic periods to 11.07% during the pandemic periods. Conversely, the standardized positivity rate increased from 0.53 to 0.62%. Our results revealed that improved test targeting and specimen collection practices were implemented for high-risk patients during the pandemic. However, our admission rate-adjusted positivity rates cannot be directly compared to unadjusted Chinese surveillance data. Notably, our institution’s unadjusted positivity rate (4.97%) was consistent with reported ranges for Chinese tertiary hospitals (4–9%) (19–21), but suggested opportunities to enhance diagnostic efficiency through optimized test indications and standardized collection protocols. Key improvement measures should include: collecting ≥2 blood culture sets (8–10 mL/bottle), optimizing collection timing, minimizing processing delays, enhancing detection of fastidious pathogens, and improving morphological identification of atypical bacteria.

Regarding the clinical characteristics of patients with positive blood cultures, more males than females and more adults than children were observed. The distribution of BSI across departments remained relatively stable, with ICUs, hematology, EICU, nephrology, and oncology departments consistently among the top five. However, the proportion of patients in the ICU, which ranked first, increased during the pandemic, suggesting that the severity of BSI in the ICU was heightened during the pandemic. Reports indicated that up to 5% of COVID-19 patients required ICU admission with more than 50% of these secondary bacterial infections, predominantly bacteremia and urinary tract infections (7, 22–24). Research has shown that most ICU-acquired blood associated with catheter-related infections, intra-abdominal infections, and ventilator-associated pneumonia (25). Therefore, it is crucial to actively treat primary diseases to prevent these complications.

The composition of BSI pathogens over the five-year period in our hospital was predominantly Gram-negative bacteria, accounting for 67.90%. This aligns closely with the 2020 national bacterial surveillance report on BSI, which reported that Gram-negative bacteria made up 73.5% of the cases (26). Some reports have indicated a trend toward an increase in the percentage of Gram-negative bacteria in BSI (27). However, the proportion of Gram-negative bacteria remained relatively stable in our hospital. The composition of BSI pathogens can vary based on factors such as time, geographic region, hospital or community setting, and patient age. In our hospital, the top three pathogens identified were *E. coli*, *K. pneumoniae*, and *S. aureus*, which aligned with the findings of the national BSI surveillance report. There were no significant changes in the detection rates of these top three pathogens before and during the pandemic. A global BSI surveillance program conducted in 2019, covering more than 200 healthcare centers, showed that the most common pathogen between 1997 and 2016 was *S. aureus*, followed by *E. coli* and *K. pneumoniae*. Notably, *S. aureus* was the most common pathogen before 2004, while *E. coli* became the most prevalent after 2005. Furthermore, the frequency of *E. coli* and *K. pneumoniae* infections increased in some regions, with the largest increases observed in Europe and Asia-Pacific (5). *S. aureus* showed a tendency of initially increasing and then decreasing over the study period (28). *K. pneumoniae*, *E. coli*, and *S. aureus* were identified as the top three pathogens responsible for deaths due to BSI (29).

Coagulase-negative staphylococci have also been reported among the top three pathogens in several regions (30, 31), which may be attributed to challenges in distinguishing strains of Coagulase-negative staphylococci, the age distribution of the population, and the classification of skin colonizers as potential pathogens. One study found that BSI caused by gram-positive bacteria were more common than those caused by Gram-negative bacteria in internal medicine wards, with *S. aureus*, coagulase-negative staphylococci, enterococci, and *Enterobacteriaceae* being the most frequently identified pathogens (32).

In our hospital, the isolation rate of *B. melitensis* and *A. baumannii* decreased during the pandemic, while the detection rates of other pathogens did not show significant changes. This highlights that the composition of pathogens in BSI is influenced by numerous factors, and local epidemiological trends should be closely monitored to inform appropriate diagnostic and treatment strategies.

The resistance rates of *E. coli*, the most common pathogen causing BSI, to amikacin, carbapenems, piperacillin/tazobactam, and cefotetan were all less than 7%. However, the resistance rate to ciprofloxacin was notably higher than 54%, and it increased during the pandemic compared to the pre-pandemic period. This suggests a growing concern over *E. coli*’s resistance to ciprofloxacin, highlighting the need for careful selection of this antibiotic in clinical practice. Additionally, the resistance rate of *E. coli* to carbapenems was found to be higher than the national bacterial resistance levels (33).

For *K. pneumoniae*, resistance rates to amikacin, carbapenems, and cefotetan were under 15%, and the resistance rate to piperacillin/tazobactam was under 20%. Despite having lower resistance rates to these antibiotics compared to *E. coli*, *K. pneumoniae* showed a relatively higher resistance profile. Notably, the resistance rate of *K. pneumoniae* to cefepime declined after the pandemic, even though there were no significant changes in the resistance rates to most other antibiotics.

*S. aureus*, the third most commonly isolated pathogen in BSI, remained sensitive to linezolid, rifampicin, tigecycline, and vancomycin, with no significant changes in its resistance rates to other antibiotics after the pandemic.

The number of multidrug-resistant bacteria causing BSI has increased over the past 5 years. However, there was no significant change in the detection rate of ESBL+ *K. pneumoniae* before and during the pandemic. In contrast, the detection rate of ESBL+ *E. coli* rose to 50.63% during the pandemic. This indicates a steady increase in ultra-broad-spectrum beta-lactamase-producing *E. coli* strains, aligning with global reports on the growing prevalence of these pathogens (5). The rising incidence of ESBL+ *Enterobacteriaceae* has become a significant concern, especially in community-acquired BSI. The detection rate of MRSA remained relatively unchanged. In a large global surveillance program, the proportion of MRSA in all *S. aureus* infections peaked a decade ago and has since declined (28). However, this downward trend has not been observed in our hospital.

The detection rate of carbapenem-resistant *A. baumannii* showed no significant change during the pandemic, even as the national detection rate decreased. Similarly, the detection rates of carbapenem-resistant *K. pneumoniae* and *E. coli* remained stable during this period. Carbapenem-resistant *Enterobacteriaceae* (CRE), known for their resistance to carbapenems and many other antibiotics, cause BSI with a higher mortality rate compared to other pathogens (34). Therefore, CRE BSI requires careful monitoring and attention. Further research into the distribution of carbapenemase enzymes in CRE strains is essential to guide the precise selection of appropriate antimicrobial therapies.

Our study showed declining trends in antimicrobial use density for total antibiotics, carbapenems, and meropenem during pandemic versus pre-pandemic, with imipenem use density slightly increasing (*p* > 0.05). No significant correlations existed between carbapenems AUD and resistance rate of *E. coli*/*K. pneumoniae* to carbapenems (*p* > 0.05), potentially due to prescribing variation, regional resistance patterns, and study duration limitations.

As a retrospective study, our analysis did not include molecular investigations of antimicrobial resistance mechanisms, which is a limitation. A further limitation involves the lack of dedicated analysis of antimicrobial resistance patterns in COVID-19 patients with BSI. These limitations highlight important directions for future research, suggesting the need for prospective studies incorporating molecular biology techniques, with particular focus on the resistance profiles in COVID-19 patients with BSI. Additionally, the primary outcomes demonstrated limited statistical significance, potentially due to: (1) inadequate sample size reducing statistical power; (2) the 5-year study duration (2018–2022) being insufficient for identifying long-term trends; and (3) pandemic-related disruptions compounded by institutional relocation in 2021. Our subsequent study will specifically address these constraints through larger cohorts, longer follow-up durations, and confounder adjustment protocols.

## 5 Conclusion

In conclusion, our study observed that the distribution of pathogens in BSI remained largely unchanged, except for a decrease in the isolation rates of *B. melitensis* and *A. baumannii* during the pandemic. The resistance of major pathogens to common antibiotics remained relatively stable when compared to the pre-pandemic period. However, the isolation rate of ESBL-producing *E. coli* increased among all multidrug-resistant bacteria. While resistance rates for certain antibiotics showed slight changes, the overall resistance situation remains concerning. For departments with a high incidence of BSI, clinicians and infection specialists should prioritize early assessment, actively manage the primary diseases, minimize exposure to risk factors, and implement multi-modal infection prevention and control strategies. Regular screening and monitoring should be conducted to guide the rational use of antibiotics and reduce the risk of BSI.

## Data Availability

The original contributions presented in the study are included in the article/supplementary material, further inquiries can be directed to the corresponding author.
